# Low Frequency Vibration Visual Monitoring System Based on Multi-Modal 3DCNN-ConvLSTM

**DOI:** 10.3390/s20205872

**Published:** 2020-10-17

**Authors:** Alimina Alimasi, Hongchen Liu, Chengang Lyu

**Affiliations:** School of Electrical and Information Engineering, Tianjin University, Tianjin 300072, China; ali_mina@tju.edu.cn (A.A.); hongchenliu@tju.edu.cn (H.L.)

**Keywords:** low frequency vibration, vibration monitoring, 3D convolutional neural network, muti-modal fusion, visual sensing

## Abstract

Low frequency vibration monitoring has significant implications on environmental safety and engineering practices. Vibration expressed by visual information should contain sufficient spatial information. RGB-D camera could record diverse spatial information of vibration in frame images. Deep learning can adaptively transform frame images into deep abstract features through nonlinear mapping, which is an effective method to improve the intelligence of vibration monitoring. In this paper, a multi-modal low frequency visual vibration monitoring system based on Kinect v2 and 3DCNN-ConvLSTM is proposed. Microsoft Kinect v2 collects RGB and depth video information of vibrating objects in unstable ambient light. The 3DCNN-ConvLSTM architecture can effectively learn the spatial-temporal characteristics of muti-frequency vibration. The short-term spatiotemporal feature of the collected vibration information is learned through 3D convolution networks and the long-term spatiotemporal feature is learned through convolutional LSTM. Multi-modal fusion of RGB and depth mode is used to further improve the monitoring accuracy to 93% in the low frequency vibration range of 0–10 Hz. The results show that the system can monitor low frequency vibration and meet the basic measurement requirements.

## 1. Introduction

Low frequency vibration generally exists in living environment and production activities. Vibrational environment has a non-negligible influence on precision instruments, bridges, buildings and human bodies [1,2,3]. Even low frequency vibration with a small amplitude in the short-term may cause or accelerate destruction. Therefore, qualitative monitoring and analysis of vibration in daily life environment is an active field to be searched.

The realization of vibration monitoring mainly contains the contact sensing mode and non-contact sensing mode. Compared with the contact sensing mode, the visual perception non-contact sensing mode [4,5,6] relies on a camcorder to record image in a timed sequence. Visual-based vibration sensing has the superiority of simple environmental requirements, non-invasion, easy operation, strong applicability and fast acquisition. With the recent developments in image processing, computer vision techniques and deep learning, the diversity and robustness of this vision-based approach has been greatly improved.

For the vibration monitoring by visual perception, how to quickly analyze the video information and realize the vibration information extraction is the core of the visual monitoring system. Traditionally, visual video analysis relies on spatiotemporal interest points described by intuitive low-level features such as SIFT [7]. Moreover, some algorithms proposed to extract single or multiple vibration points in the frame images. Jiantao Liu et al. [8] proposed image sequence analysis by reading video as an image sequence and saving it as separate pixel brightness vibration signals. Our previous research [9] studied the projected color fringe from a vibrating plane and chose a center point of a series of images to record the surface height changes for gain vibration frequency. U.P. Poudel, G et al. [10] used digital video imaging for detecting damaging in the structures, which is based on sub-pixel edge identification to obtain the time series of a vibrating object. According to the characteristics of vibration in a time domain, feature engineering of these methods transforms two-dimensional information into one-dimensional information to realize the vibration measuring or monitoring. The extracted one-dimensional information is a biased representation of the original signal sequence, not a perfect representation in the overall view of vibration. In fact, the vibration is not only correlated in temporal and one-dimensional vibration information structure does not completely cover the spatial characteristics of vibration. One-dimensional signal sequence can only represent vibration information in which every independent variable corresponds to only one dependent variable. Image as a first-hand obtained information carrier of morphological features and changes of spatial and temporal information records the vibration in space. The complicated structure distribution and interrelationship is one where every independent variable may have more than one dependent variable. We hope to put the two-dimensional information of a vibration video directly into the network, so as to represent the whole vibration change.

The main purpose of feature engineering is to reduce the modeling complexity by reducing the input dimension. The complete characteristics of a video stream in space is not easy to recognize from manual feature engineering. However, the advances in modeling methods have made it possible to directly take high dimensional data as the model input. Currently, given the interest in learning deep with its strong self-learning ability and dependence on getting rid of manual intervention and expert experience, convolutional neural networks (CNN) have pretty good performances in many scopes [11,12,13]. Several vibration research methods combining with it in some stage have been proposed recently. Ruoyu Yang et al. [14] selected the deep learning CNN-LSTM approach as a backbone to serve the computer vision-based vibration measurement techniques. Jiantao Liu et al. [15] proposed image-based machine learning via LSTM-RNNs combined with a multi-target learning techniques method to measure the vibration frequency. Huipeng Chen et al. [16] combined the two-direction vibration bearing signal data with a deep convolutional neural network for fault diagnosis. Compared with traditional methods, deep learning has a strong feature extraction ability for a large amount of complex data automatically with the serial structure of feature extractor and classifier. The improvement in the neural network makes it possible to take the vibration video (original high dimensional signal) as the input to directly estimate the essential relationship between the input and the vibration states, which may release researchers from constrained manual feature engineering. In theory, without manual feature engineering, the model is the best relationship representation in mathematics, rather than the empirical model based on signal-feature-model method.

Based on the RGB camera, there have been some attempts to detect small objects with the help of geometry cues and CNNs. However, when the vibration environment such as ambient light is not stable, the vibration information collected may be affected. Relying on apparent information in the RGB image alone is not sufficient. It is necessary to obtain multi-sided vibration information to describe the vibration at each moment, so at least one mode auxiliary to the RGB mode is required. The depth mode contains more location, contour and spatial information that can be used as a critical indicator of objects. To achieve more comprehensive and accurate results, the fusion of modes is essential [17,18,19]. The data level fusion is mainly used to integrate signals such as the various types of video resources, which is a low-level fusion. It has a high demand for data homogeneity, and poor real-time performance. Feature level fusion fuses information after feature extraction. Data compression in feature extraction is considerable, but there may be information loss in fusion. Decision level fusion is based on different sensors to obtain the target object processing, which has strong flexibility, a low requirement for data homogeneity and a strong fault tolerance, but the learning ability could be limited. Combined with the characteristics of modal and convenience in practical operation, it is necessary and significant to adopt a method that can intelligently and effectively give the results.

In this work, we propose a method supported by depth mode and RGB mode information acquired from Microsoft Kinect v2 and combine it with 3D convolutional networks and conventional LSTM (3DCNN-ConvLSTM), which is a further development for monitoring the vibration frequency.

The main contributions of this paper are summarized as follows:

- We propose to use 3D convolution to extract spatial and temporal features from the vibration video stream in the field of vibration monitoring for the first time. Compared with the traditional vibration feature extraction method, the feature extractor operates in both spatial and temporal dimensions to capture the vibration features from raw data automatically without depending on the signal processing techniques in the video stream.
- We implement the network corresponding to vibration signal characteristics to realize frequency classification and monitoring. In the low frequency vibration range, the 3DCNN and ConvLSTM network architecture can effectively learn the spatial-temporal characteristics of muti-frequency with both global and local features. 3DCNN is used to extract the short-term spatiotemporal feature. The ConvLSTM structure learns the long-term spatiotemporal feature information.
- The method we propose is non-invasive and has no special restrictions on the monitoring environment. In order to reduce the interference factors such as ambient light and meet more comprehensive vibration monitoring as far as possible, we set the depth mode as the auxiliary of the color mode, and improve the performance of vibration monitoring through multi-modal fusion. The experimental results show that the method is superior to the single-modal structure.

The remainder of this paper is organized as follows. In Section 2, materials and methods of the system are described. In Section 3, experiment evaluation of different objects is made to verify the effectiveness and superiority of the proposed method. After this, the obtained results with the discussions and the conclusions are given in Section 4.

## 2. Materials and Methods

**Figure 1:**
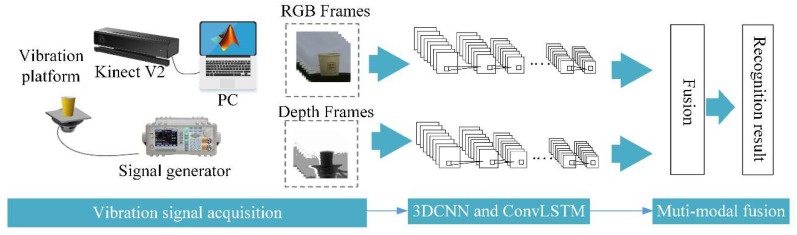
Flowchart of the proposed method.

In this paper, a multi-modal (depth and RGB) visual vibration monitoring system based on Kinect v2 and 3DCNN-ConvLSTM is proposed. The procedure of the novel method to realize vibration recognition is shown in Figure 1. There are three main parts: vibration signal acquisition, 3DCNN-LSTM and multi-modal fusion. In this method, the depth mode of Kinect v2 are complementary to the RGB mode to collect the input visual information. The input of the network is the collected sample without the tedious feature extraction processing. According to a fusion formula in the late stage the process of fusion multi-modal is carried out.

### 2.1. Vibration Signal Acquisition 

RGB-D camera has been a rising technology in recent years [20,21]. Kinect v2, a common RGB-D camera, can simultaneously record the vibration video of objects in both RGB mode and depth mode. The depth mode of Kinect is based on a flight (TOF) algorithm. The basic principle of TOF is to continuously transmit light pulses (generally invisible light) to the observed object and calculates the distance between the sensor and the object based on the flight time. Compared with color image, depth image contains depth information indicating the actual distance from the sensor to the object. The interference of background light is reduced to a certain extent due to the high energy of the light pulses. Theoretically when the vibration occurs, the depth images have superior advantages with the texture-less and sharp-edge characteristics. In the process of acquisition, there is no need for strict adjustment or camera calibration and only collecting the image of the vibrating object within the view is needed. Owing to these characteristics, depth information can be complemented to RGB mode for the monitoring of the whole vibration process.

Vibration signal acquisition is shown in Figure 1. We use a signal generator to provide sinusoidal signals with a frequency from 0 Hz to 10 Hz and peak to peak voltage 20 V to control the vibration platen. The object to be monitored is placed in the platen. The acquisition equipment Kinect v2 is used to take vibration frames of RGB and depth modes. The frame rate of two mode is 30 fps. In the end, a series of RGB and depth mode frame images are obtained, which records the object vibration process in a low frequency vibration environment.

### 2.2. Networks for Vibration Signal 

**Figure 2:**
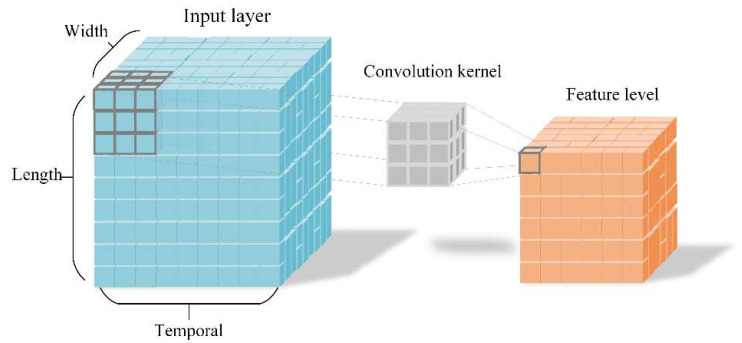
Process representation of 3DCNN.

In order to learn the vibration feature in the space, it is desirable to capture the vibration information encoded in contiguous frames. In Figure 2, the video frame information is represented by blue squares with three dimensions including width, length and temporal. The three-dimensional convolution kernel will perform slide window operations in height, width and temporal directions on video stream. The image receptive field of the input layer convolutes by three-dimensional convolution kernel, the temporal features can be extracted at one time, and the state change information of multiple frames can be captured. However, when 2D convolution operating is in process, there is no relation between the extracted feature in the temporal direction. After the sum operator, all features are collapsed. Compared with the two-dimensional convolution principle, three-dimensional convolution [22,23] with its pooling could be performed spatiotemporally.

Formally, the value of a unit at position (x,y,z) in the jth feature map in the ith layer, denoted as $a_{ij}^{xyz}$, is given by

(1) $$a_{ij}^{xyz}=f[b_{ij}+\sum_{p=0}^{P_{i}-1}\sum_{q=0}^{Q_{i}-1}\sum_{r=0}^{R_{i}-1}w_{ij}^{pqr}a_{i-1}^{\left(x+p)(y+q)(z+r\right)}]$$

where $P_{i}$ is the length of the 3D kernel, $Q_{i}$ is the width of the 3D kernel and $R_{i}$ is the size of the 3D kernel along the temporal dimension. $w_{ij}^{pqr}$ is the weight of 3D kernel position (p, q, r) connecting the upper image receptive field. $b_{ij}$ represents the bias and f(x) represents the activation function.

**Figure 3:**
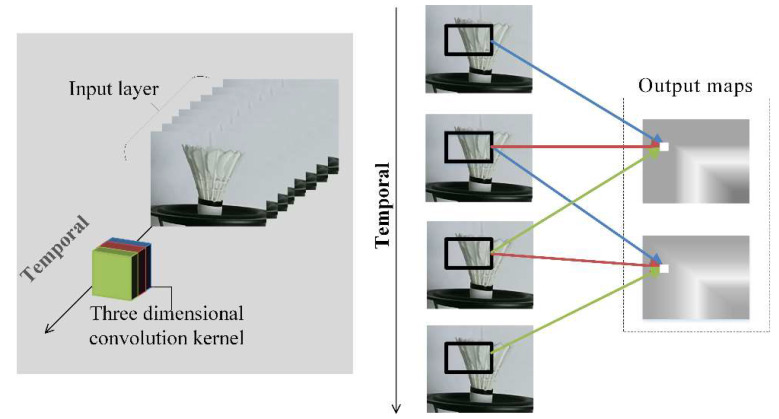
Feature with a kernel from the RGB video stream in temporal.

In Figure 3, the 3D convolution kernel with length 3 (red, green and blue) in the temporal direction executes two times convolution operations sequentially in the temporal direction to obtain output maps. The weight of the convolution kernel is the same in the whole video stream and one convolution kernel can only extract one type of feature. The features pointed by the different color arrows to the same output map containing temporal information, and all output maps form a 3D tensor. So the 3D convolutional network is well-suited for the vibration feature learning in frame images. 

**Figure 4:**
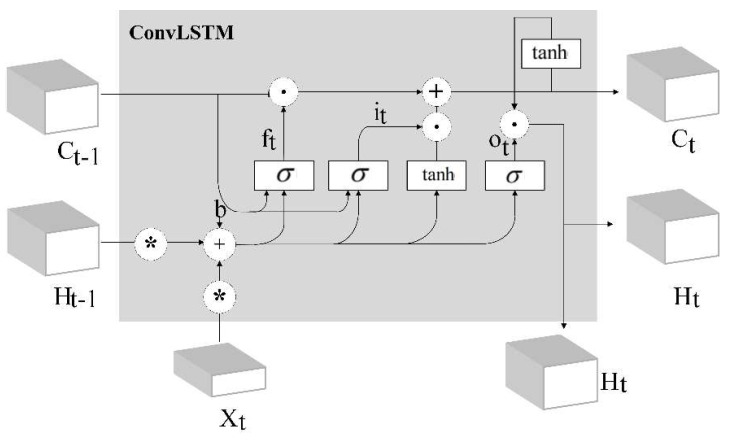
The structure of convolution LSTM.

3DCNN operates in a comparatively short-term spatiotemporal space, and applying 3DCNN architectures alone to the time-series related problem is sub-optimal. Long short-term memory (LSTM) networks are skillful in sequential learning by passing signal information across time steps [14]. As shown in Figure 4, when the input vector $X_{t}$ is fed as 3-D matrices after the 3DCNN, ConvLSTM [24] could be considered. ConvLSTM replaces the convolution operators with an LSTM memory cell. ConvLSTM has been applied in a time-series classification for anomaly detection using video sequences.

The ConvLSTM can be formulated as

(2) $$i_{t}=\sigma (W_{xi}*X_{t}+W_{ci}*H_{t-1}+W_{ci}*C_{t-1}+b_{i}),$$

(3) $$f_{t}=\sigma (W_{xf}*X_{t}+W_{hf}*H_{t-1}+W_{cf}\circ C_{t-1}+b_{f})$$

(4) $$C_{t}=f_{t}\circ C_{t-1}+i_{t}\circ \tanh(W_{xc}*X_{t}+W_{hc}\circ H_{t-1}+b_{c}),$$

(5) $$o_{t}=\sigma (W_{xo}*X_{t}+W_{ho}*H_{t-1}+W_{co}\circ C_{t}+b_{o}),$$

(6) $$H_{t}=o_{t}\circ \tanh(C_{t})$$

where $X_{1},X_{2},\ldots ,X_{i-1},X_{i},X_{i+1}$ are the inputs, $C_{1},C_{2},\ldots ,C_{i-1},C_{i},C_{i+1}$ are the cell states, $H_{1},H_{2},\ldots ,H_{i-1},H_{i},H_{i+1}$ are the hidden states and $f_{t},i_{t},C_{t},o_{t}$ are the gates.σ is the sigmoid function and W is the weight corresponding to different state gates. The symbol ‘◦’represents the multiplication of the corresponding elements of the matrix, also known as the Hadamard product.

3DCNN-ConvLSTM architecture uses 3D Convolutional Neural Network layers coupled with ConvLSTM for feature extraction on input spatio-temporal characterization of vibration data.

### 2.3. Model of Muti-Modal Networks 

**Figure 5:**
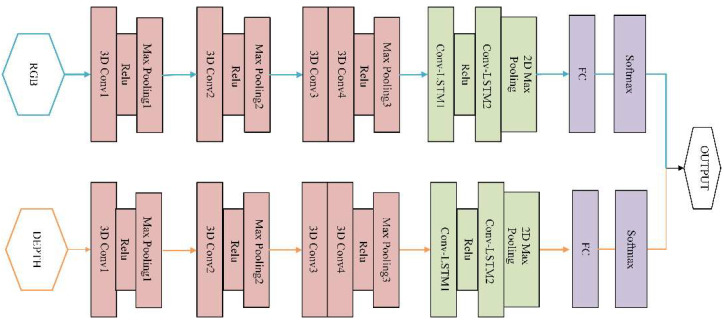
An architecture of muti-modal fusion model.

The model design should match the characteristics of vibration learning spatiotemporal features simultaneously. In Figure 5, an architecture with small 3 × 3 × 3 convolution kernels in all layers is among the best performing architecture for 3DCNN. The learning ability is positively related to the number of layers and the size of the kernels. If the structure is too simple, the learning ability will be so poor that it cannot effectively integrate the vibration information with the small amplitude. Table 1 shows the details of the 3DCNN-Convlstm used in the model. The filter counts of the four Conv3D layers are 30, 60, 80, 80 respectively according to the complexity of the data. Relu is a nonlinear activation function which improves the distinguishability of the learned features.

The ConvLSTM obtains the temporal information between frames based on the spatial information extracted by 3DCNN. Two-level ConvLSTM is deployed in the proposed algorithm. The convolutional kernel size is 3 × 3 with stride 1 × 1. The convolutional filter counts of the two-level ConvLSTM layers are 256 and 384, respectively. The output of the high level ConvLSTM layer is down-sampled by 2D max pooling, and then flattened into a 1D tensor, which is considered as the long-term spatiotemporal features for each vibration frequency. This 1D tensor is then fully connected to 11 nodes by the fully connected (FC) layer. After the softmax layer, the probability of each class is obtained.

**Figure 6:**
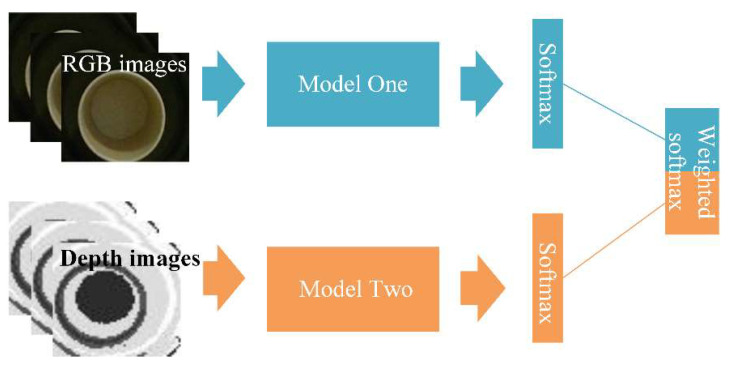
Schematic diagram of multi-modal fusion.

The schematic diagram of multi-modal fusion is shown in Figure 6. The softmax layer is used to calculate the probability distribution of each class. The formula of softmax could be expressed as follows:

(7) $$P_{i}{=\;\mathrm{softm}\;\mathrm{ax}(x}_{i})=\frac{e^{x_{i}}}{\sum_{j}e^{x_{j}}}$$

where x is the output vector of the full connection layer and has the dimension the same as the number of classification categories and $x_{i}$ is the element value of vector x. $P_{i}$ is the softmax value $x_{i}\;$ of representing the probability that this input is class i. The output vectors of the two softmax layers are the predicted probabilities of each class by two modal networks. Then the mean of the two prediction vectors is calculated to get the prediction vector. Combining the two scores according to fusion Formula (8):

(8) $$P=Average(P_{RGB}+P_{DEPTH})$$

P is the final prediction vector, $P_{RGB}$ and $P_{DEPTH}$ are the prediction vectors of RGB and depth mode respectively.

## 3. Experiments

### 3.1. Experimental Setup and Dataset Description

**Figure 7:**
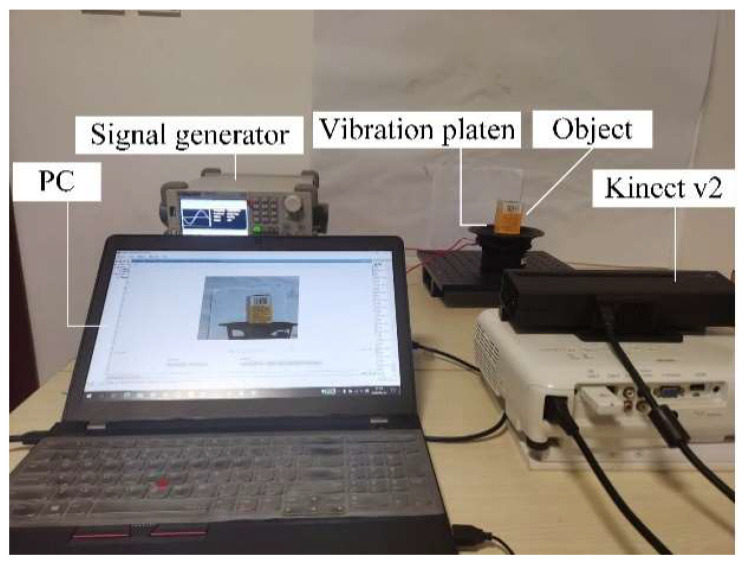
Experimental structure and environment.

**Figure 8:**
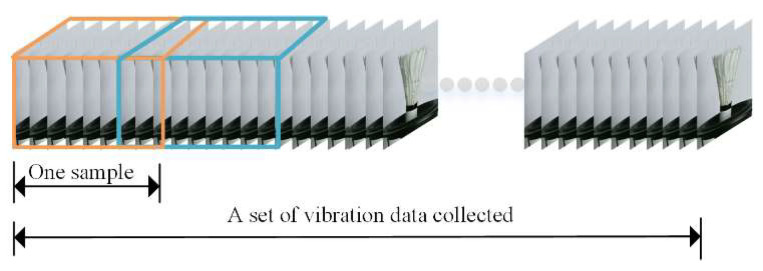
Data augment and preparation.

Our dataset is composed of videos recording objects in a low-frequency vibration environment. The experimental dataset is obtained using the device and sample shown in Figure 7. The vibration platen is controlled by a certain frequency sinusoidal waves generated by the signal generator. The monitored object is placed on a vibration platen which provides up and down vibration. We control Microsoft Kinect v2 sensor through MATLAB SDK toolkit to capture RGB and depth vibration videos of the different objects. The system can be placed in different positions to obtain images from any arbitrary perspective without complicated calibration process. The Regions-of-Interest (ROIs) of RGB and the depth information are selected for the monitoring area consistent with 259 × 213 px. The camera frame rate is 30 fps and thus we could capture 1500 frames of vibration videos in 50 s. Figure 8 shows the data augmentation and preparation process. The raw vibration image signals are sliced with overlap, which helps increase the amount of training data. An integer between 60 and (1500–60) is randomly selected as the sampling starting point, and 60 consecutive frames are selected as a vibration sample. 20 samples are selected by this means. Each sample (the different color block) is used as an input segment to construct the 3DCNN-ConvLSTM.

For our experiment, datasets of 3 vibrating objects of 11 vibration frequencies were collected in contrast ambient lights, which is a total of 66 videos for RGB and depth modes. Twenty samples were extracted from each video, thus a total of 2640 video samples were obtained. They represent a total of 11 annotations (labels). The RGB dataset is divided into a training set and a test set according to the proportion of 70% and 30% (Table 2) and the depth dataset is allocated in the same way. Train dataset as input to train the model and test dataset is used to evaluate the effectiveness of the trained model. We used 10-fold cross validation for evaluation. The samples were randomly divided into 10 samples, 9 of which were used as training set and the remaining one as verification set. After repeating 10 cycles, we used the model with the highest average accuracy of ten validation sets as the test model.

### 3.2. Experiments and Results

In our work, we have compared our model introduced in Section 2.3 with a single branch model either using RGB data or depth data. Moreover, we have compared the performance between our proposed network with 3DCNN.

Due to the deep network structure and the large input data dimension, the required memory space for training these models is indeed far greater, necessitating a reduction in the batch size for training neural networks. We set the batch size to 4 in the experiment. The number of epoch is set to 550. The initial learning rate is set as 0.001. The decay point of the RGB mode is carried on the training 300 epoch. Depth mode of the decay point is carried on the training 430 epoch. The networks are implemented based on the Keras platforms and trained from scratch. These experiments were carried out on an Intel Xeon e5-2620 v4 CPU@2.10 on servers (Dell, Xiamen, China) with 2.10 GHz, 128 GB RAM, GeForce GTX Titan XP and CentOS Linux 7.6.

**Figure 9:**
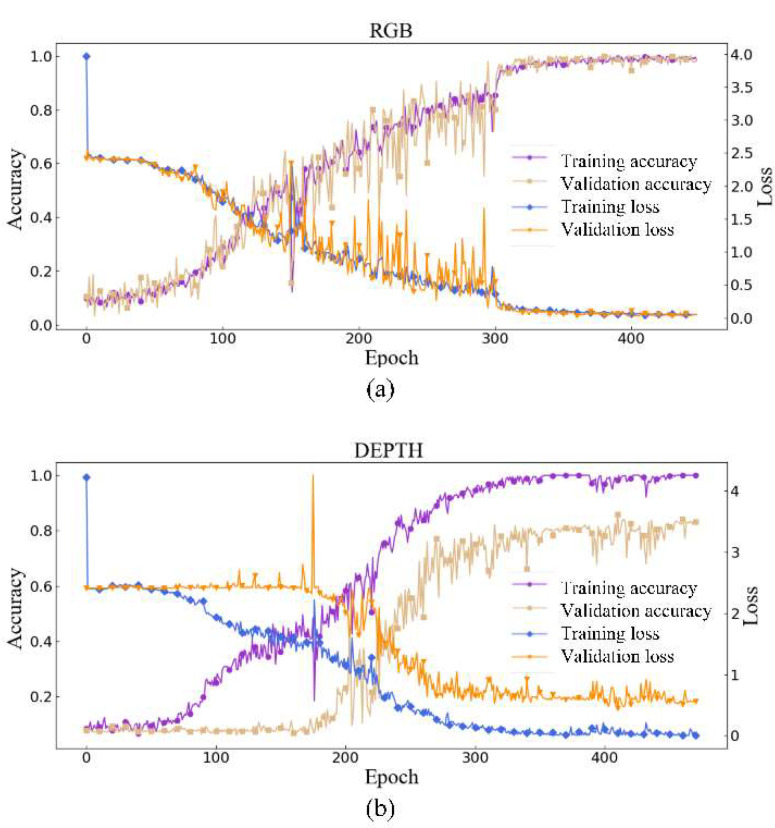
Training process of RGB/depth mode. (**a**) RGB mode of training process; (**b**) depth mode of training process.

The training process for of the RGB mode (a) and depth mode (b) is shown in Figure 9. The RGB model and the depth model were trained separately. During training we used cross-entropy loss and accuracy as two evaluating indicators. Loss value is used to evaluate the difference between the output of the network and the true label value. Accuracy is the ratio of the number of correct samples out of the total samples. The validation (Val) set is used to help adjust the training process. As is shown in Figure 9, the accuracy and loss for RGB and depth mode tend to be stable after 300 to 350 epochs. Compared with the RGB mode, the depth mode still has larger accuracy jitter after 300 epochs. We think this is because the depth camera in Kinect v2 has a lower resolution and precision than the RGB camera. The performance on validation sets also proves that the learned features of 11 distinct kinds of frequency vibration signals are effective on two modes.

As it can be seen in Table 3, we select the model with the highest validation accuracy for the RGB mode and the depth mode. In the test, the RGB and depth mode perform 89% and 82%, respectively. The accuracy reaches 93% for detection and classification in videos with the multi-modal fusion, which outperforms either of single modes. Our goal to make the depth mode complementary to RGB is achieved in this fusion method.

In order to better evaluate the performance between the fusion and the single mode models, four result measurements are calculated to quantify the performance, namely the mean accuracy, recall, precision and F1-score. The formulas of the four evaluation indexes can be seen in Equations (9)–(12). True positive (TP) is correctly classified as positive samples, false positive (FP) is misclassified as positive samples, true negative (TN) is correctly classified as negative samples, and false negative (FN) is misclassified as negative samples.

(9) $$\mathrm{Recall}=\frac{TP}{TP+FN}$$

(10) $$Accurancy=\frac{TP+TN}{TP+FP+FN+TN}$$

(11) $$Precision=\frac{TP}{TP+FP}$$

(12) $$F1-score=\frac{2\times \mathrm{Precision}\times \mathrm{Recall}}{\mathrm{Precision}+\mathrm{Recall}}$$

Evaluation indicator results of the fusion mode and RGB mode are shown in Table 4. As can be seen from the data in the table, the average values of precision, recall and F1-score all increase by 4%. For the 9 Hz class, the precision is increased from 85% to 100% and the difference between precision and recall becomes smaller as well. For example, the difference between precision and recall of 8 Hz class for the RGB mode equals to 26%, while the difference is only 4% for the fusion mode, which proves the fusion model has more balanced and reliable prediction.

**Figure 10:**
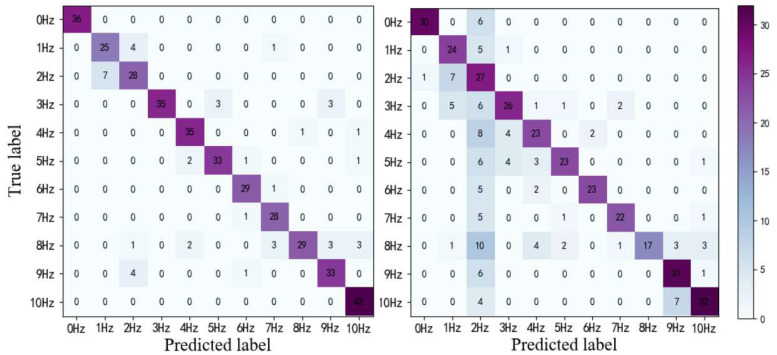
Confusion matrixes. (**a**) 3DCNN-CONVLSTM; (**b**) 3DCNN.

In order to verify the advantages of the ConvLSTM network in vibration measurements, we compare the difference between the proposed model with the 3DCNN without ConvLSTM. We train the two models to converge in the same RGB dataset. The confusion matrix results of the experiments are displayed in Figure 10. The F1-score of the 3DCNN-ConvLSTM network is 89%, while that of 3DCNN is 72%. The thermal map of the confusion matrix also proves that the model is superior to the only model based on 3DCNN.

We think the low inter-class variability of datasets makes the task with temporal information more difficult for 3DCNN. LSTM is naturally better at learning the characteristics of time domain. Thus, based on the spatial information extracted by 3DCNN-ConvLSTM could perform much better in this scene.

## 4. Discussion and Conclusions

The context of the presented work is the challenging task of monitoring low frequency vibration in non-contact video methods. To meet that aim, we have proposed an approach for a low frequency vibration visual monitoring system based on multi-modal 3DCNN-ConvLSTM. Based on the experiments conducted with different objects vibrating at different frequencies in the above-mentional experimental environment, the proposed method can provide acceptable vibration monitoring result which reaches an accuracy of 93%.

Compared with the traditional image-based methods, our method does not need extra image processing or signal transformation, and directly uses the collected images to put into the network for model training and testing. This could provide great benefits for real-time vibration detection. At the same time, the construction and deployment of the whole hardware is fast and convenient. Due to the characteristics of vibration recorded by frame images, we use an RGB-D camera to add modal information to record the vibration spatial information, which further improves the accuracy of vibration monitoring.

Visual information can be obtained quickly and easily, especially based on the performance improvement of hardware devices. This means that the visual vibration monitoring method can be widely used in precision instruments, human health, bridge monitoring and other aspects. Moreover, we can consider transplanting this method to portable equipment to monitor the low frequency vibration environment.

We still acknowledge the drawbacks and our next research may focus on the next two aspects. (1) enriching the vibration data in a variety of complex scenarios and gradually improve the robustness of the network model. (2) reducing the complexity of the network while maintaining the detection accuracy, so that the model can run faster or run on ordinary performance devices.

## Figures and Tables

**Table 1:** Details of the 3DCNN-ConvLSTM used in the model.

Layer	Parameters
3D Conv1	KW = 3; KH = 3; kL = 3; KC = 3 (1 for depth mode); KN = 30; stride = 1 × 1 × 1
Max Pooling1	PS = 1 × 2 × 2; stride = 1 × 2 × 2
3D Conv2	KW = 3; KH = 3; kL = 3; KC = 30; KN = 60; stride = 1 × 1 × 1
Max Pooling2	PS = 1 × 2 × 2; stride = 2 × 2 × 2
3D Conv3	KW = 3; KH = 3; kL = 3; KC = 60; KN = 80; stride = 1 × 1 × 1
3D Conv4	KW = 3; KH = 3; kL = 3; KC = 80; KN = 80; stride = 1 × 1 × 1
Max Pooling 3	PS = 2 × 2 × 2; strides = 1 × 1 × 1
ConvLSTM1	KW = 3; KH = 3; KC = 80; KN = 256; stride = 1 × 1
ConvLSTM2	KW = 3; KH = 3; KC = 256; KN = 384; stride = 1 × 1
2D Max Pooling	PS = 7 × 7; stride = 7 × 7
FC	Nodes = 11

KW = kernel width; KH = kernel height; KL = kernel length; KC = kernel channel; KN = number of kernels in the convolution layer; PS = pool window size.

**Table 2:** RGB Datasets.

Datasets	Train	Test	Summary
Object 1 (Dixie cup)	310	130	440
Object 2 (Badminton)	314	126	440
Object 3 (Box)	299	141	440
Summary	923	397	1320

**Table 3:** Performance comparison of the different models. (The best performance is depicted in bold).

Models	Accuracies (%)	
	Val ^1^	Test
RGB (one branch)	99.8	89.0
DEPTH (one branch)	83.0	82.0
Multi-modal fusion	—	**93.0**

^1^ The accuracy of validation set for the model selected.

**Table 4:** Evaluation indicator results of the muti-modal fusion and RGB mode.

Category	Precision		Recall		F1-Score	
	Fusion	RGB	Fusion	RGB	Fusion	RGB
0 Hz	1.00	1.00	1.00	1.00	1.00	1.00
1 Hz	0.79	0.78	0.90	0.83	0.84	0.81
2 Hz	0.89	0.76	0.89	0.80	0.89	0.78
3 Hz	1.00	1.00	0.83	0.85	0.91	0.92
4 Hz	0.92	0.90	0.89	0.95	0.90	0.92
5 Hz	0.92	0.92	0.95	0.89	0.93	0.90
6 Hz	0.97	0.91	1.00	0.97	0.98	0.94
7 Hz	0.94	0.85	1.00	0.97	0.97	0.90
8 Hz	0.86	0.97	0.90	0.71	0.88	0.82
9 Hz	1.00	0.85	0.92	0.87	0.96	0.86
10 Hz	0.98	0.90	1.00	1.00	0.99	0.95
Average	0.93	0.89	0.93	0.89	0.93	0.89
